# High Prevalence of Sarcopenia in Noncirrhotic Women With Primary Biliary Cholangitis: A Comparative Study of Clinical Characteristics and Risk Factors

**DOI:** 10.1155/grp/8688776

**Published:** 2026-05-30

**Authors:** Jingyi Zhang, Jiamin Xu, Weimin Bao, Yingmei Tang

**Affiliations:** ^1^ Department of Gastroenterology, The Second Affiliated Hospital of Kunming Medical University, Kunming, Yunnan, China, kmmc.cn; ^2^ Department of General Surgery, Yunnan Provincial First People′s Hospital, Kunming, Yunnan, China

**Keywords:** female, primary biliary cholangitis, risk factors, sarcopenia

## Abstract

**Aim:**

Sarcopenia is a common complication in patients with chronic liver disease (CLD). However, most prior studies on sarcopenia in CLD have predominantly focused on viral hepatitis and cirrhosis, with limited data available on its prevalence in primary biliary cholangitis (PBC), especially at noncirrhotic stages. This study is aimed at investigating the clinical characteristics and identifying independent risk factors for sarcopenia in female PBC patients.

**Methods:**

We retrospectively enrolled 229 female patients, including 118 with chronic hepatitis B (CHB) and 111 with PBC. All patients underwent abdominal computed tomography (CT). The skeletal muscle area at the level of the third lumbar vertebra (L3) was measured using the image analysis software SliceOmatic V5.0 (TomoVision, Magog, Canada), and the skeletal muscle index (SMI) was calculated accordingly. Sarcopenia was defined based on the L3‐SMI index. We compared the prevalence of sarcopenia between PBC and CHB patients at different stages of liver fibrosis and further analyzed the clinical characteristics and independent risk factors for sarcopenia in PBC patients.

**Results:**

Among the 229 female patients, 93 were noncirrhotic (45 with PBC and 48 with CHB). The prevalence of sarcopenia was significantly higher in noncirrhotic PBC patients than in noncirrhotic CHB patients (75.60% vs. 31.30%, *χ* ^2^ = 18.29, *p* < 0.001). Among the 136 cirrhotic patients (66 with PBC and 70 with CHB), sarcopenia prevalence showed no significant difference between PBC and CHB patients (60.60% vs. 52.90%, *χ* ^2^ = 0.83, *p* > 0.05). After propensity score matching (PSM), both cirrhotic and noncirrhotic PBC groups demonstrated a high prevalence of sarcopenia (85.30% vs. 70.60%, *χ* ^2^ = 2.138, *p* > 0.05). Multivariate analysis identified that low body mass index (BMI) (OR = 1.342; 95*%*CI = 1.150–1.565; *p* < 0.001) and osteopenia/osteoporosis (OR = 4.2; 95*%*CI = 1.408–12.533; *p* = 0.01) were independent risk factors for sarcopenia in female PBC patients.

**Conclusion:**

Sarcopenia is highly prevalent in female PBC patients. In contrast to female CHB patients, in whom sarcopenia mainly occurs at the cirrhotic stage, female PBC patients exhibit high sarcopenia prevalence at both noncirrhotic and cirrhotic stages. Low BMI and osteopenia/osteoporosis are independent risk factors for sarcopenia in female PBC patients. Notably, sarcopenia should also be considered in PBC patients with normal or high BMI. Early and routine screening for sarcopenia and its risk factors is recommended in all PBC patients, regardless of cirrhosis stage.

## 1. Introduction

Sarcopenia is a chronic, progressive syndrome characterized by the loss of skeletal muscle mass and strength. Primary sarcopenia is predominantly associated with advancing age [1, 2]. In contrast, secondary sarcopenia is a frequent and serious complication in patients with chronic liver disease (CLD), with the highest prevalence observed in those with cirrhosis. Advanced age, male sex, female menopause, and systemic inflammation are established risk factors for sarcopenia [3–5]. The reported incidence of sarcopenia in cirrhotic patients ranges from 30% to 70% [6], varying with the underlying etiology of liver disease. Cirrhotic patients with sarcopenia exhibit significantly increased risks of hepatic decompensation and are more susceptible to complications such as hepatic encephalopathy, refractory ascites, and spontaneous bacterial peritonitis [7–11]. These patients experience a reduced quality of life and higher mortality [12]. As a well‐established cause of cirrhosis, chronic hepatitis B (CHB) has been extensively studied, and previous studies have shown a significantly elevated incidence of sarcopenia in the cirrhotic stage of the disease [13].

Primary biliary cholangitis (PBC) is an autoimmune, cholestatic liver disease characterized by the progressive destruction of small‐to‐medium intrahepatic bile ducts [14], predominantly affecting middle‐aged women [15]. Beyond hepatic injury, PBC is frequently associated with various extrahepatic manifestations, including osteoporosis and dyslipidemia, as well as multisystem autoimmune disorders such as autoimmune thyroiditis, systemic lupus erythematosus, and Sjögren′s syndrome [16, 17]. The systemic inflammatory response and abnormal bone metabolism resulting from these complications can both adversely affect muscle metabolism [18, 19]. Collectively, these pathophysiological features suggest that the prevalence and clinical presentation of sarcopenia in PBC may be distinct from those in other CLDs.

However, current research on sarcopenia in PBC remains limited. This study focused on female patients, who represent the predominant PBC population, to eliminate gender‐related confounding factors associated with sarcopenia development. By comparing the prevalence of sarcopenia between PBC and CHB patients at noncirrhotic and cirrhotic stages, this study is aimed at exploring the clinical characteristics and risk factors of sarcopenia in PBC patients, thereby providing a reference for the clinical management of this population.

## 2. Materials and Methods

### 2.1. Participants

We retrospectively enrolled 229 female patients with CLD who were treated at the Department of Hepatology, The Second Affiliated Hospital of Kunming Medical University from January 2018 to July 2020. The study cohort was divided into two diagnostic subgroups: 118 patients with CHB and 111 with PBC. All patients underwent abdominal computed tomography (CT) scans.

The inclusion criteria were as follows: (1) PBC patients meeting the diagnostic standards of the European Association for the Study of the Liver (EASL) Clinical Practice Guidelines: Diagnosis and Management of PBC patients [20]; and (2) CHB patients meeting the EASL Clinical Practice Guidelines on the management of hepatitis B virus infection [21].

The exclusion criteria included: (1) male patients; (2) coinfected with other hepatitis viruses, metabolic dysfunction‐associated fatty liver disease, or drug‐induced liver disease; (3) comorbid hepatocellular carcinoma or other malignancies, hyperthyroidism, or other hypermetabolic diseases; and (4) pregnant or lactating women.

The study protocol conformed to the ethical guidelines of the Declaration of Helsinki and was approved by the Ethics Committee of the Second Affiliated Hospital of Kunming Medical University (IRB‐PJ‐2020‐96). Given the retrospective nature of the study, the ethics committee waived the requirement for informed consent. All patient identifiers were removed or encrypted to protect confidentiality and privacy. All data were analyzed after aggregation and anonymization to further protect patient information.

### 2.2. Diagnosis of Sarcopenia

All patients underwent abdominal CT scans (Siemens, 256‐slice, Germany). CT was selected over dual‐energy X‐ray absorptiometry (DXA) as it is widely recognized as the gold standard for body composition analysis and is less influenced by fluid overload, a common comorbidity in liver disease patients. The cross‐sectional skeletal muscle area at the level of the third lumbar vertebra (L3) was measured on CT images using SliceOmatic software (V5.0 Rev‐9, TomoVision, Canada). Skeletal muscle tissue was identified using Hounsfield unit (HU) thresholds ranging from −29 to +150. The measured skeletal muscle area included the psoas major, erector spinae, transversus abdominis, quadratus lumborum, rectus abdominis, internal abdominal oblique, and external abdominal oblique, which together constitute the L3 skeletal muscle area (L3‐SMA). The L3 skeletal muscle index (L3‐SMI) was calculated as the skeletal muscle area (cm^2^) divided by height squared (m^2^) and expressed as cm^2^/m^2^. Sarcopenia was defined as an L3‐SMI < 38 cm^2^/m^2^ in female patients, in accordance with the diagnostic criteria established by the Japanese Society of Hepatology [22].

### 2.3. Diagnosis of Osteopenia and Osteoporosis

Patients′ bone mineral density (BMD) was assessed using DXA (GE LUNAR, GE Healthcare 13.31, United States). The diagnosis of osteopenia/osteoporosis was based on the World Health Organization (WHO) 2004 criteria [23]: (1) Normal BMD: *T*‐score > −1.0; (2) osteopenia: *T*‐score between −2.5 and −1.0; and (3) osteoporosis: *T*‐score ≤ −2.5.

### 2.4. Collection of Clinical Data

Patient data were systematically extracted from the hospital′s electronic medical record system, including the following categories: (1) General information: age, gender, menstrual history, and body mass index (BMI); (2) clinical characteristics: liver disease duration, medication history, symptoms of cirrhotic decompensation, and comorbidities (e.g., diabetes and hypertension); (3) laboratory indicators: serum alanine aminotransferase (ALT), aspartate aminotransferase (AST), alkaline phosphatase (ALP), gamma‐glutamyl transferase (GGT), albumin (ALB), total bilirubin (TB), total bile acid (TBA), blood urea nitrogen (BUN), serum calcium (Ca^2+^), total cholesterol (TC), triglycerides (TG), high‐density lipoprotein (HDL), low‐density lipoprotein (LDL), immunoglobulin A/G/M (IgA, IgG, IgM), complement 3/4 (C3,C4), interleukin‐6 (IL‐6), and autoantibodies (anti‐SSA, anti‐SSB, AMA‐M2, anti‐gp210, and anti‐sp100); and (4) imaging data: abdominal CT and DXA BMD examination.

### 2.5. Statistical Analysis

Statistical analyses were performed using SPSS (Version 23.0; IBM Corp, Armonk, New York, United States). Data normality was assessed using the Shapiro–Wilk test. Normally distributed continuous variables were expressed as mean ± standard deviation and compared using independent‐samples *t*‐tests. Nonnormally distributed continuous variables were expressed as median (P25 and P75) and analyzed using Mann–Whitney *U* tests. Categorical variables were expressed as frequencies (%), with between‐group comparisons conducted using chi‐square tests or Fisher′s exact test, with Bonferroni correction for multiple comparisons; ordinal categorical variables were compared using the Wilcoxon rank‐sum test. Multivariable analysis was performed using binary logistic regression analysis. Propensity score matching (1:1 ratio) was implemented with a caliper width of 0.02. A two‐tailed *p* value < 0.05 was considered statistically significant.

## 3. Result

### 3.1. Characteristics of Patients

A total of 229 female patients with CLD were enrolled in this study. Among them, 111 patients had PBC. The PBC cohort was stratified into noncirrhotic (*n* = 45) and cirrhotic (*n* = 66) subgroups. Noncirrhotic PBC patients had a mean age of 52.00 ± 10.97 years, 28 (62.20%) were menopausal, 7 (15.60%) had hypertension, and none had diabetes or fractures. Cirrhotic PBC patients had a mean age of 59.06 ± 1.20 years, 56 (84.80%) were menopausal, 21 (31.80%) had hypertension, 11 (16.70%) had diabetes, and 7 (10.60%) had fractures. The remaining 118 patients were diagnosed with CHB. The CHB cohort comprised 48 noncirrhotic and 70 cirrhotic patients. Noncirrhotic CHB patients had a mean age of 47.38 ± 13.38 years, 21 (43.80%) were menopausal, 6 (12.50%) had hypertension, 4 (8.30%) had diabetes, and 1 (2.10%) had fractures. Cirrhotic CHB patients had a mean age of 55.64 ± 1.28 years, 55 (78.60%) were menopausal, 17 (24.30%) had hypertension, 8 (11.40%) had diabetes, and 3 (4.30%) had fractures (Tables 1 and 2).

**Table 1:** Comparison of clinical characteristics and prevalence of sarcopenia between noncirrhotic patients with PBC and CHB.

Variable	Noncirrhotic CHB *n* = 48	Noncirrhotic PBC *n* = 45	T/Z/*χ* ^2^ value	*p* value
Age (years)	47.38 ± 13.38	52 ± 10.97	−1.816	0.073
Menopausal status (%)	21 (43.80)	28 (62.20)	3.179	0.075
Hypertension (%)	6 (12.50)	7 (15.60)	0.18	0.671
Diabetes (%)	4 (8.30)	0 (0)	2.155	0.142
Fracture (%)	1 (2.10)	0 (0)	0	1
Sarcopenia (%)	15 (31.30)	34 (75.60)	18.29	< 0.001
ALT (U/L)	39 (19.25, 140)	42 (23, 116.50)	−0.461	0.645
AST (U/L)	36.50 (21, 114.50)	48 (26, 100.50)	−0.927	0.354
ALP (U/L)	77.50 (64.75, 97.50)	145 (109, 200)	−5.428	< 0.001
GGT (U/L)	31 (15.25, 90.25)	103 (61.50, 189)	−4.613	< 0.001
ALB (g/L)	40.15 (34.10, 43.55)	39.40 (36.15, 44.60)	−0.292	0.77
TB (umol/L)	14.5 0(10.13, 20.73)	17.90 (12.25, 34.85)	−1.257	0.209

Abbreviations: ALB, albumin; ALP, alkaline phosphatase; ALT, alanine aminotransferase; AST, aspartate aminotransferase; CHB, chronic hepatitis B; GGT, gamma‐glutamyl transferase; PBC, primary biliary cholangitis; TB, total bilirubin.

**Table 2:** Comparison of clinical characteristics and prevalence of sarcopenia between cirrhotic patients with PBC and CHB.

Variable	Cirrhotic CHB *n* = 70	Cirrhotic PBC *n* = 66	T/Z/*χ* ^2^ value	*p* value
Age (years)	55.64 ± 1.28	59.06 ± 1.20	−1.943	0.054
Menopausal status (%)	55 (78.60)	56 (84.80)	0.892	0.345
Hypertension (%)	17 (24.30)	21 (31.80)	0.957	0.328
Diabetes (%)	8 (11.40)	11 (16.70)	0.776	0.379
Fracture (%)	3 (4.30)	7 (10.60)	1.172	0.279
Sarcopenia (%)	37 (52.90)	40 (60.60)	0.83	0.362
ALT (U/L)	28 (20, 54)	38 (22, 69.50)	−1.342	0.18
AST (U/L)	42 (31, 64)	58 (33, 95.75)	−1.903	0.057
ALP (U/L)	114 (82, 148)	199.50 (114.50, 323.25)	−4.943	< 0.001
GGT (U/L)	30 (16.50, 68)	98.50 (32.50, 179.50)	−4.488	< 0.001
ALB (g/L)	34.28 ± 6.29	33.00 ± 6.90	1.033	0.304
TB (umol/L)	21.90 (14.15, 41.85)	28.20 (15.65, 77.18)	−1.624	0.104
Child–Pugh classification			−0.965	0.335
Child–Pugh A (%)	26 (37.10)	27 (40.90)		
Child–Pugh B (%)	22 (31.40)	25 (37.90)		
Child–Pugh C (%)	22 (31.40)	14 (21.20)		

Abbreviations: ALB, albumin; ALP, alkaline phosphatase; ALT, alanine aminotransferase; AST, aspartate aminotransferase; CHB, chronic hepatitis B; GGT, gamma‐glutamyl transferase; PBC, primary biliary cholangitis; TB, total bilirubin.

### 3.2. Comparison of the Occurrence of Sarcopenia in Noncirrhotic Patients

A total of 93 female patients with CLD at the noncirrhotic stage were included, consisting of 45 noncirrhotic PBC patients and 48 noncirrhotic CHB patients. No significant differences existed between the two groups in terms of age, menopausal status, histories of hypertension, diabetes, and fractures, as well as levels of ALT, AST, ALB, and TB (*p* > 0.05). Noncirrhotic PBC patients had significantly higher levels of ALP and GGT compared with noncirrhotic CHB patients (*p* < 0.001). Sarcopenia was diagnosed in 15 (31.30%) noncirrhotic CHB patients versus 34 (75.60%) noncirrhotic PBC patients, indicating a significantly higher prevalence of sarcopenia in the latter group (*χ* ^2^ = 18.29, *p* < 0.001) (Table 1).

### 3.3. Comparison of the Occurrence of Sarcopenia in Cirrhotic Patients

A total of 136 female patients with liver cirrhosis were included, consisting of 66 cirrhotic PBC patients and 70 cirrhotic CHB patients. There were no significant differences between the two groups in terms of age, menopausal status, histories of hypertension, diabetes, or fractures, as well as Child–Pugh liver function classification or levels of ALT, AST, ALB, and TB (*p* > 0.05). Cirrhotic PBC patients had significantly higher levels of ALP and GGT than cirrhotic CHB patients (*p* < 0.05). Sarcopenia was diagnosed in 37 (52.90%) cirrhotic CHB patients versus 40 (60.60%) cirrhotic PBC patients, showing no significant difference in prevalence between the two groups (*χ* ^2^ = 0.83, *p* > 0.05) (Table 2).

### 3.4. Comparison of Clinical Characteristics Between PBC Patients With Sarcopenia and Those Without

A total of 111 female PBC patients were included. Of these, 74 (66.70%) were diagnosed with sarcopenia, and 37 (33.30%) were nonsarcopenic. Comparing the two groups, the prevalence of osteopenia and osteoporosis was significantly higher in the sarcopenic group than in the nonsarcopenic group (*p* < 0.05). Patients with sarcopenia had a significantly lower BMI compared with those without (*p* < 0.05). There were no significant differences in age or menopausal status between the two groups (*p* > 0.05). The rates of response to ursodeoxycholic acid (UDCA) treatment, hormone use, diuretics use, and transjugular intrahepatic portosystemic shunt (TIPS) treatment showed no significant differences between the groups (*p* > 0.05). Furthermore, histories of hypertension, diabetes, fractures, other extrahepatic autoimmune diseases, as well as the prevalence of cirrhosis, splenomegaly, portal hypertension, ascites, hepatic encephalopathy, and gastrointestinal bleeding showed no significant differences (*p* > 0.05). Finally, there were no significant differences in autoimmune‐related antibody profiles, biochemical indicators, or Child–Pugh classification between the two groups (*p* > 0.05) (Table 3).

**Table 3:** Clinical characteristics of PBC patients with and without sarcopenia.

Variable	Sarcopenia *n* = 74	Nonsarcopenia *n* = 37	T/Z/*χ* ^2^ value	*p* value
Age (years)	57.10 ± 10.66	54.41 ± 10.97	−1.241	0.217
Menopausal status (%)	59 (79.70)	27 (73)	0.645	0.422
Hypertension (%)	17 (23)	11 (29.70)	0.597	0.44
Diabetes (%)	10 (13.50)	1 (2.70)	2.132	0.144
UDCA treatment response (%)	44 (59.50)	21 (56.80)	0.074	0.785
Hormone use (%)	23 (31.10)	13 (35.10)	0.185	0.667
Diuretics use (%)	8 (10.80)	0 (0)	2.846	0.092
Other extrahepatic autoimmune disease (%)	31 (41.90)	13 (35.10)	0.471	0.493
Osteopenia and osteoporosis (%)	66 (89.20)	24 (64.90)	9.514	0.002
Splenomegaly (%)	46 (62.20)	25 (67.60)	0.313	0.576
Portal hypertension (%)	33 (44.60)	17 (45.90)	0.018	0.893
Hepatic encephalopathy (%)	3 (4.10)	1 (2.70)	< 0.001	1
Ascites (%)	22 (29.70)	13 (35.10)	0.334	0.563
Gastrointestinal bleeding (%)	9 (12.20)	3 (8.10)	0.105	0.746
TIPS treatment (%)	9 (12.20)	2 (5.40)	0.618	0.432
Cirrhosis (%)	40 (54.10)	26 (70.30)	2.691	0.101
Positive anti‐SSA (%)	20 (27)	10 (27)	< 0.001	1
Positive anti‐SSB (%)	5 (6.80)	2 (5.40)	< 0.001	1
Positive AMA‐M2 (%)	42 (56.80)	24 (64.90)	0.673	0.412
Positive anti‐gp210 (%)	25 (33.80)	12 (32.40)	0.02	0.887
Positive anti‐sp100 (%)	7 (9.50)	4 (10.80)	<0.001	1
BMI	20.48 (18.64, 22.52)	24.14 (22.24, 25.39)	−4.943	< 0.001
ALB (g/L)	35.75 (28.35, 40.43)	37.60 (30.15, 41.80)	−1.289	0.197
ALT (U/L)	39 (23.75, 71.25)	42 (19, 94)	−0.225	0.822
AST (U/L)	60.50 (30.75, 102.50)	52 (27.50, 117.50)	−0.454	0.65
ALP (U/L)	181.5 (123.50, 280.75)	154 (95, 243)	−1.298	0.194
GGT (U/L)	103 (58.75, 206.25)	88 (24, 173.50)	−1.451	0.147
TB (umol/L)	22.50 (12.75, 49.60)	20.70 (15.70, 64.90)	−0.563	0.573
TBA (umol/L)	50.60 (15.63, 120.68)	49.60 (11.50, 143.70)	−0.31	0.757
BUN (mmol/L)	53 (48, 63)	57 (51, 67.50)	−1.615	0.106
TC (mmol/L)	4.27 (3.26, 5.57)	4.39 (3.58, 5.54)	−0.488	0.626
TG (mmol/L)	1.29 (1.01, 1.69)	1.39 (0.96, 1.84)	−0.31	0.757
HDL (mmol/L)	1.24 ± 0.66	1.35 ± 0.69	0.82	0.414
LDL (mmol/L)	2.37 (1.94, 3.35)	2.69 (1.86, 3.45)	−0.535	0.593
Ca^2+^ (mmol/L)	2.19 ± 0.20	2.19 ± 0.18	< 0.001	1
IgG (mmol/L)	16.60 (13.58, 22.33)	17.10 (14, 20.45)	−0.041	0.968
IgA (g/L)	3.04 (2.31, 4.09)	3.22 (2.45, 4.45)	−0.651	0.515
IgM (g/L)	2.42 (1.41, 3.24)	2.62 (1.57, 3.70)	−0.751	0.453
C3 (g/L)	0.83 ± 0.28	0.83 ± 0.23	0.068	0.946
C4 (g/L)	0.17 (0.13, 0.23)	0.18 (0.13, 0.26)	−0.376	0.707
IL‐6 (pg/mL)	11.28 (3.58, 15.82)	12.94 (4.99, 15.95)	−0.968	0.333

Abbreviations: ALB, albumin; ALP, alkaline phosphatase; ALT, alanine aminotransferase; AST, aspartate aminotransferase; AMA‐M2, anti‐mitochondrial antibody‐M2; anti‐SSA, Sjögren′s syndrome‐related antigen A; anti‐SSB, Sjögren′s syndrome‐related antigen B; BMI, body mass index; BUN, blood urea nitrogen; Ca^2+^, serum calcium; C3, complement 3; C4, complement 4; GGT, gamma‐glutamyl transferase; gp210, anti‐glycoprotein 210; HDL, high‐density lipoprotein; IgA, immunoglobulin A; IgG, immunoglobulin G; IgM, immunoglobulin M; IL‐6, interleukin‐6; LDL, low‐density lipoprotein; PBC, primary biliary cholangitis; sp100, anti‐speckled protein 100; TB, total bilirubin; TBA, total bile acid; TC, total cholesterol; TIPS, transjugular intrahepatic portosystemic shunt; TG, triglyceride; UDCA, ursodeoxycholic acid.

### 3.5. Factors Associated With Sarcopenia in Female PBC Patients

In the univariate analysis, only two variables were significantly associated with sarcopenia: the presence of osteopenia/osteoporosis and low BMI. Subsequently, these variables were incorporated into a multivariate binary logistic regression model to identify independent predictors of sarcopenia. The results showed that the presence of osteopenia/osteoporosis (OR = 4.2; 95*%*CI = 1.408–12.533; *p* = 0.01) and low BMI (OR = 1.342; 95*%*CI = 1.15–1.565; *p* < 0.001) were identified as independent risk factors for sarcopenia in female PBC patients (Table 4).

**Table 4:** Binary logistic regression analysis of factors associated with sarcopenia in female PBC patients.

Variable	*B*‐coefficient	Standard error	Wald *χ* ^2^	*p* value	Odds ratio	95% confidence interval for odds ratio	
						Lower	Upper
Osteopenia/osteoporosis	1.435	0.558	6.620	0.010	4.200	1.408	12.533
BMI (per 1 reduction)	0.294	0.079	13.951	< 0.001	1.342	1.150	1.565

Abbreviation: BMI, body mass index.

### 3.6. Prevalence Comparison of Sarcopenia Between Cirrhotic PBC Patients and Noncirrhotic PBC Patients

Among the 111 PBC patients (66 cirrhotic and 45 noncirrhotic), there were significant differences in age, menopausal status, histories of diabetes, osteopenia/osteoporosis, and levels of ALP, ALB, and TB between the two groups (*p* < 0.05). No significant differences were found in BMI, history of hypertension, or levels of ALT, AST, and GGT (*p* > 0.05). The prevalence of sarcopenia in noncirrhotic PBC patients showed no significant difference compared with cirrhotic PBC patients (75.60% vs. 60.60%, *χ* ^2^ = 2.691, *p* > 0.05).

Patients were matched at a 1:1 ratio using propensity score matching (PSM), resulting in 34 noncirrhotic and 34 cirrhotic PBC patients. No significant differences were found in age, menopausal status, histories of hypertension, diabetes, osteopenia/osteoporosis, or levels of ALT, AST, ALP, and GGT between the matched groups (*p* > 0.05). Cirrhotic PBC patients had significantly lower levels of ALB and higher levels of TB than noncirrhotic PBC patients (*p* < 0.05). After PSM, sarcopenia remained highly prevalent in both noncirrhotic and cirrhotic PBC patients, and no significant difference in its prevalence persisted between the matched groups (85.30% vs. 70.6%, *χ* ^2^ = 2.138, *p* > 0.05) (Table 5).

**Table 5:** Comparison of clinical characteristics between noncirrhotic and cirrhotic PBC patients in the overall and PSM cohorts.

Variables	Overall cohort				PSM cohort			
	Noncirrhotic PBC *n* = 45	Cirrhotic PBC *n* = 66	T/Z/*χ* ^2^ value	*p* value	Noncirrhotic PBC *n* = 34	Cirrhotic PBC *n* = 34	T/Z/*χ* ^2^ value	*p* value
Age (years)	52 ± 10.97	59.06 ± 9.75	−3.559	0.001	55.88 ± 8.30	56.29 ± 8.23	−0.205	0.838
Menopausal status (%)	30 (66.70)	56 (84.80)	5.069	0.024	28 (82.40)	27 (79.40)	0.095	0.758
Hypertension (%)	7 (15.60)	21 (31.80)	3.752	0.053	7 (20.60)	11 (32.40)	1.209	0.272
Diabetes (%)	0	11 (16.70)	6.563	0.004	0	4 (11.80)	2.391	0.122
Osteopenia/osteoporosis (%)	30 (66.70)	60 (90.90)	10.251	0.001	29 (85.30)	29 (85.30)	< 0.001	1
BMI	21.76 (18.63, 23.37)	21.99(19.97, 24.16)	−0.898	0.369	21.16 (18.58, 23.25)	22.13 (20.00, 23.55)	−1.055	0.291
ALT (U/L)	42 (23, 116.50)	38 (22, 69.50)	−0.811	0.417	40.50 (23, 114)	41 (22.75, 71.25)	−0.337	0.736
AST (U/L)	48 (26, 100.50)	63 (33, 105.25)	0.94	0.347	48.50 (26, 116.25)	66.50 (33.75, 102.50)	−1.079	0.28
ALP (U/L)	145 (109, 200)	199.5 0(114.50, 323.25)	−2.276	0.023	144.50 (118.25, 196)	195.50 (121.75, 290.75)	−1.595	0.111
GGT (U/L)	103 (61.50, 189)	98.50 (32.50, 179.50)	−0.73	0.466	104.50 (65, 182)	94.50 (28, 240)	−0.803	0.422
ALB (g/L)	39.40 (36.15, 44.60)	32.70 (27.30, 40.03)	−4.082	< 0.001	39.15 (35.58, 45.15)	33.40 (27.23, 37.18)	−3.367	0.001
TB (umol/L)	19.90 (12.25, 34.85)	28.20 (15.65, 77.18)	−2.793	0.005	15.50 (11.88, 34.98)	31.80 (15.48, 77.35)	−2.508	0.012
DB (umol/L)	6.50 (3.85, 26.05)	15.05 (6.50, 63.18)	−3.346	0.001	5.55 (3.65, 27.50)	15.05 (6.68, 63.33)	−3.288	0.001
Sarcopenia (%)	34 (75.60)	40 (60.60)	2.691	0.101	29 (85.30)	24 (70.60)	2.138	0.144

Abbreviations: ALB, albumin; ALP, alkaline phosphatase; AST, aspartate aminotransferase; ALT, alanine aminotransferase; BMI, body mass index; GGT, gamma‐glutamyl transferase; DB, direct bilirubin; PBC, primary biliary cholangitis; PSM, propensity score matching; TB, total bilirubin.

## 4. Discussion

Our study focused exclusively on female PBC patients to exclude the confounding effect of the increased risk of sarcopenia associated with male patients. The average age of the enrolled patients was 56.20 ± 10.97 years. When comparing the prevalence of sarcopenia between PBC and CHB patients with cirrhosis, there was no significant difference (60.60% vs. 52.90%, *χ* ^2^ = 0.83, *p* = 0.362). However, among noncirrhotic patients, the prevalence of sarcopenia was significantly higher in PBC patients than in CHB patients (75.60% vs. 31.30%, *χ* ^2^ = 18.29, *p* < 0.001). Further analysis revealed that PBC patients had a high prevalence of sarcopenia across both noncirrhotic and cirrhotic stages (85.30% vs. 70.60%, *χ* ^2^ = 2.138, *p* = 0.144). These results suggest that, while sarcopenia in CLD is mainly associated with cirrhosis, in PBC patients, its prevalence is elevated throughout the disease course.

Previous studies have shown that the prevalence of sarcopenia in patients with cirrhosis and end‐stage liver disease is 30%–70%, and it is more common in male patients with a male‐to‐female ratio of approximately 2:1, findings that are consistent with our results. The mechanisms underlying sarcopenia in cirrhotic patients include inadequate nutritional intake, endocrine disorders, inflammatory responses, and reduced physical activity [24, 25]. Cirrhosis leads to weakened gastrointestinal function, decreased appetite, and insufficient nutritional intake, failing to meet the normal metabolic demands of muscles. Impaired liver metabolism results in abnormalities in glucose and protein metabolism; gluconeogenesis, which supplies energy to muscles, is diminished, and the liver′s capacity to synthesize proteins is reduced, leading to decreased muscle protein synthesis and accelerated breakdown [26]. Additionally, impaired hepatic hormone inactivation leads to dysregulation of the growth hormone‐insulin‐like growth factor 1 (GH‐IGF‐1) axis. IGF‐1 is critical for muscle growth and repair; its downregulation directly inhibits muscle protein synthesis, impeding muscle growth and repair [12]. Testosterone promotes muscle protein synthesis in skeletal muscle cells; in cirrhotic patients, decreased hepatic synthesis of testosterone increases the risk of muscle atrophy in males [27]. Simultaneously, the cirrhotic stage is characterized by elevated systemic inflammatory responses and oxidative stress. Inflammatory cytokines activate catabolic signaling pathways for muscle proteins, while oxidative stress exacerbates mitochondrial damage in muscle cells. These processes can initiate apoptotic signaling pathways within muscle cells, leading to apoptosis [28, 29]. Cirrhotic patients often experience declining physical activity due to reduced strength and mobility impairment, particularly when complicated by ascites, contributing to muscle atrophy [30]. In hyperammonemia, liver dysfunction and portal‐systemic shunting diminish the detoxification capacity for ammonia. Skeletal muscles play a compensatory role in ammonia metabolism and clearance [31]. The increased demand for branched‐chain amino acids (BCAAs) in muscle ammonia metabolism depletes the BCAAs required for muscle protein synthesis, thereby reducing muscle synthesis [32]. Concurrently, high ammonia concentrations in skeletal muscle induce mitochondrial dysfunction, increase reactive oxygen species formation, and promote oxidative stress, ultimately leading to oxidative damage of muscle proteins and lipids, contributing to sarcopenia [29, 33]. In our study, all patients were female to mitigate the influence of testosterone levels on muscle mass. PBC predominantly affects middle‐aged women. The decline in estrogen levels following menopause in these patients can contribute to a reduction in skeletal muscle mass. We assessed and controlled for the menopausal status across patient groups to account for its potential influence on sarcopenia prevalence, thereby enhancing the precision and reliability of our research fundings [34].

Multivariate binary logistic regression analysis revealed that comorbid osteopenia/osteoporosis is an independent risk factor for sarcopenia in PBC patients. Previous studies have shown that osteopenia/osteoporosis is also a common complication of CLD. The incidence of osteoporosis in PBC patients is as high as 20%–52%, significantly higher than in patients with other CLDs. As the disease progresses, only 20% of patients with advanced PBC maintain normal bone mass [35]. PBC patients can develop osteopenia/osteoporosis in both noncirrhotic and cirrhotic stages [36]. The reasons are twofold. Firstly, most PBC patients are middle‐aged women, and both advanced age and declining estrogen levels postmenopause are established risk factors for osteoporosis [37, 38]. Secondly, autoimmune inflammation in PBC leads to elevated levels of bilirubin, bile acids, and inflammatory factors such as IL‐6, all of which contribute to the increased incidence of osteopenia/osteoporosis [39, 40]. Bone and muscle share similar genetic backgrounds, molecular signaling pathways, and are influenced by common metabolic (endocrine and paracrine) factors [41, 42]. Thus, operating under these shared mechanisms, osteopenia/osteoporosis is strongly associated with the development of sarcopenia. Saeki et al. [43] demonstrated that osteoporosis was an independent risk factor for sarcopenia in PBC patients, a finding consistent with our present study. The prevalence of sarcopenia among female PBC patients in our study was higher than that reported by Saeki et al., which may be attributed to several factors. Firstly, different ethnic backgrounds and regional variation in dietary habits and lifestyles may influence patients′ skeletal muscle mass. Secondly, the different methods employed for diagnosing sarcopenia represent another significant factor. In our study, sarcopenia was diagnosed using the L3‐SMI derived from CT images. The L3‐SMA was measured using specialized software, and the L3‐SMI was calculated by normalizing L3‐SMA to height squared. In contrast, Saeki′s study diagnosed sarcopenia based on handgrip strength and bioelectrical impedance analysis (BIA)–derived skeletal muscle mass. BIA estimates muscle mass from body water conduction and impedance, which can overestimate muscle mass in patients with fluid retention. Furthermore, BIA‐based indices normalized by weight can be confounded by ascites. In comparison, the CT method is considered the gold standard for measuring skeletal muscle mass [1].

It should be noted that this study used CT‐based L3‐SMI to assess sarcopenia radiologically. While this method can accurately quantify muscle mass and is minimally affected by fluid overload in patients with liver disease, it cannot evaluate muscle strength or physical function directly. Current consensus guidelines issued by both the European and Asian Sarcopenia Working Groups recommend a sequential assessment paradigm involving screening tools, along with evaluation of muscle strength, muscle mass, and physical function, as the gold standard for sarcopenia diagnosis [1, 44]. This paradigm integrates patients′ muscle function with clinical indicators, thereby yielding a more comprehensive diagnostic outcome. In the clinical management of PBC patients, CT‐based assessment facilitates the screening of individuals with pre‐existing abdominal imaging data. However, in clinical practice, a comprehensive diagnosis still necessitates adherence to consensus guidelines integrated with functional indicators. Given that this is a retrospective study, we are unable to obtain relevant data such as patient screening tools, muscle strength measurements, and physical function assessment. Nevertheless, CT‐based L3‐SMI enabled accurate measurement of muscle mass, a method that is widely utilized in sarcopenia‐related scientific research [12]. Thus, this study remains an important reference for the preliminary exploration of sarcopenia in PBC patients.

Furthermore, the findings of this study confirm that a low BMI is another independent risk factor for sarcopenia in PBC patients. Malnutrition is present in 65%–90% of cirrhotic patients, and BMI serves as a general indicator for evaluating the nutritional status in PBC patients [45, 46]. Poor nutritional status is closely associated with the development of sarcopenia. In cirrhotic PBC patients, impaired liver function can lead to disturbances in the absorption and anabolic metabolism of carbohydrates, lipids, and proteins. Even in the noncirrhotic stage, chronic cholestasis may impair the absorption of lipids and fat‐soluble vitamins like vitamin D [47]. Collectively, these factors elicit nutritional deficiencies, which ultimately exert detrimental impacts on muscle metabolism. Timely identification and remediation of malnutrition may effectively prevent the development of sarcopenia in PBC patients. Notably, a normal or even elevated BMI cannot fully exclude the presence of sarcopenia. Recent studies have indicated that approximately 20% of patients with CLD present with sarcopenic obesity. These patients exhibit a substantially higher risk of adverse clinical outcomes, with significantly higher Model for End‐Stage Liver Disease (MELD) scores, risk of liver decompensation and mortality rates, compared with CLD patients who have simple obesity or simple sarcopenia [48]. Although BMI is easy to calculate and remains widely utilized in clinical practice for evaluating nutritional status, this index neither distinguishes between muscle mass and fat mass, nor does it account for the confounding interference of pathological edema and ascites that commonly occur in CLD patients. Consequently, sarcopenia may still exist in PBC patients despite a normal or elevated BMI. This suggests that body composition assessment should be prioritized in the clinical management of PBC patients. Not only should patients with low BMI undergo intensive screening, but those with normal or elevated BMI should also undergo a comprehensive evaluation to identify potential cases of occult sarcopenic obesity.

Besides the aforementioned risk factors, the potential impact of treatment response on sarcopenia also warrants in‐depth investigation. Existing studies have indicated that approximately 40% of PBC patients exhibit a suboptimal response to UDCA, the first‐line treatment of PBC [49]. In the present study, no significant difference was observed in the UDCA treatment response rate between PBC patients with and without sarcopenia (59.50% vs. 56.80%, *χ* ^2^ = 0.074, *p* = 0.785). Nevertheless, the incidence of UDCA nonresponse remained at a relatively high level in both groups, at 40.5% and 43.2%, respectively. Such nonresponders generally require combination therapy or a switch to second‐line therapeutic agents, and the myotoxicity of certain second‐line agents, as well as their potential effects on bone and muscle metabolism deserve particular attention. Among the clinically commonly used second‐line treatment regimens for PBC, the off‐label use of benzafibrate and fenofibrate has been reported to induce myalgia and muscle weakness in approximately 5%–20% of treated patients, with severe cases even leading to rhabdomyolysis [50, 51]. Several lines of evidence suggest that these fibrates may exacerbate unrecognized pre‐existing myopathies in patients [52]. Elafibranor, a novel dual peroxisome proliferator‐activated receptor *α*/*δ* (PPAR*α*/*δ*) agonist, has been approved by the U.S. Food and Drug Administration (FDA) and the European Medicines Agency (EMA) for the treatment of UDCA‐refractory PBC. Of clinical concern, emerging evidence indicates that elafibranor has been associated with an increased risk of bone fracture and reduced BMD in some patients. Furthermore, and very rare cases of elevated creatine kinase levels and rhabdomyolysis have been observed in a small subset of patients receiving elafibranor, implying its potential adverse impact on bone and muscle metabolism in PBC patients [53]. Therefore, for PBC patients who fail to respond to UDCA therapy, the evaluation of muscle function and sarcopenia status should be prioritized when selecting second‐line treatments. For patients with already existing sarcopenia, intensified monitoring of muscle health (e.g., regular detection of creatine kinase, assessment of muscle strength, muscle mass, and physical function) is recommended during medication administration. Alternatively, agents with confirmed myotoxicity should be avoided whenever possible, so as to mitigate the risk of treatment‐related muscle damage and improve the overall prognosis of PBC patients.

Our study provides an in‐depth exploration of the prevalence and clinical characteristics of sarcopenia between noncirrhotic and cirrhotic female PBC patients, and comparing these findings with those from another CLD etiology. The results indicate that, unlike female CHB patients, who primarily develop sarcopenia during the cirrhotic stage, female PBC patients exhibit a high prevalence of sarcopenia in both noncirrhotic and cirrhotic stages. The coexistence of osteopenia/osteoporosis and a low BMI are identified as independent risk factors for sarcopenia in this population. Consequently, the management of PBC patients should emphasize early and routine screening for sarcopenia and its risk factors throughout the entire disease course, to facilitate timely nutritional and therapeutic interventions that may improve muscle mass and overall prognosis.

**Nomenclature**
PBC: primary biliary cholangitis
CHB: chronic hepatitis B
CT: computed tomography
L3: third lumbar vertebra
SMA: skeletal muscle area
SMI: skeletal muscle index
BMI: body mass index
OR: odds ratio
EASL: European Association for the Study of the Liver
BMD: bone mineral density
DXA: dual‐energy X‐ray absorptiometry
WHO: World Health Organization
ALT: alanine aminotransferase
AST: aspartate aminotransferase
ALP: alkaline phosphatase
GGT: gamma‐glutamyl transferase
ALB: albumin
TB: total bilirubin
TBA: total bile acid
BUN: blood urea nitrogen
Ca^2+^
: serum calcium
TC: total cholesterol
TG: triglycerides
HDL: high‐density lipoprotein
LDL: low‐density lipoprotein
IgA: immunoglobulin A
IgG: immunoglobulin G
IgM: immunoglobulin M
C3: complement 3
C4: complement 4
IL‐6: interleukin‐6
AMA‐M2: anti‐mitochondrial antibody M2
anti‐gp210: anti‐glycoprotein 210
anti‐sp100: anti‐speckled protein 100
UDCA: ursodeoxycholic acid
TIPS: transjugular intrahepatic portosystemic shunt

## Author Contributions

**Jingyi Zhang:** writing – review & editing, writing – original draft, validation, project administration, methodology, investigation, formal analysis, data curation. **Jiamin Xu:** writing – review & editing, formal analysis, data curation. **Weimin Bao:** writing – review & editing, data curation. **Yingmei Tang:** writing – review & editing, supervision, project administration, methodology, funding acquisition, formal analysis, data curation, conceptualization.

## Funding

This study was supported by National Natural Science Foundation of China (81660102 and 82360108) and Yunnan Fundamental Research Projects) (202601CI070206).

## Ethics Statement

This study was conducted in accordance with the ethical guidelines of the Declaration of Helsinki. The study protocol was reviewed and approved by the Ethics Committee of the Second Affiliated Hospital of Kunming Medical University (IRB‐PJ‐2020‐96). Due to the retrospective nature of the study and the use of anonymized patient data, the requirement for informed consent was waived by the ethics committee. All patient identifiers were removed or encrypted to ensure confidentiality and privacy. Data were analyzed in an aggregated and anonymized form to further protect patient information.

## Conflicts of Interest

The authors declare no conflicts of interest.

## Data Availability

The data supporting this study are available from the corresponding author upon reasonable request, subject to privacy and ethical restrictions.

## References

[1] Cruz-Jentoft A. J. , Bahat G. , Bauer J. , Boirie Y. , Bruyère O. , Cederholm T. , Cooper C. , Landi F. , Rolland Y. , Sayer A. A. , Schneider S. M. , Sieber C. C. , Topinkova E. , Vandewoude M. , Visser M. , Zamboni M. , and Writing Group for the European Working Group on Sarcopenia in Older People 2 (EWGSOP2), and the Extended Group for EWGSOP2 , Sarcopenia: Revised European Consensus on Definition and Diagnosis, Age Ageing. (2019) 48, no. 4, 10.1093/ageing/afz046, 2-s2.0-85069484790, 31081853. PMC6593317 31081853

[2] He N. , Zhang Y. L. , Zhang Y. , Feng B. , Zheng Z. , Wang D. , Zhang S. , Guo Q. , and Ye H. , Circulating MicroRNAs in Plasma Decrease in Response to Sarcopenia in the Elderly, Frontiers in Genetics. (2020) 11, 10.3389/fgene.2020.00167, 32194634. PMC7066121 32194634

[3] Xiao H. J. , Ye Q. , Zhang M. , Qi Y. M. , Han T. , and Wang X. , Risk Factors for Sarcopenia and its impacts on Clinical outcome in patients with liver cirrhosis, Chinese Journal of Hepatology. (2020) 28, no. 1, 53–57. 32023700 10.3760/cma.j.issn.1007-3418.2020.01.013 PMC12770240

[4] Tandon P. , Ney M. , Irwin I. , Ma M. M. , Gramlich L. , Bain V. G. , Esfandiari N. , Baracos V. , Montano-Loza A. J. , and Myers R. P. , Severe Muscle Depletion in Patients on the Liver Transplant Wait List: Its Prevalence and Independent Prognostic Value, Liver Transplantation. (2012) 18, no. 10, 1209–1216, 10.1002/lt.23495, 2-s2.0-84867519687, 22740290. 22740290

[5] Ponziani F. R. and Gasbarrini A. , Sarcopenia in Patients With Advanced Liver Disease, Current Protein and Peptide Science. (2018) 19, no. 7, 681–691, 10.2174/1389203718666170428121647, 2-s2.0-85048888245. 28462693

[6] Mazurak V. C. , Tandon P. , and Montano-Loza A. J. , Nutrition and the Transplant Candidate, Liver Transplantation. (2017) 23, no. 11, 1451–1464, 10.1002/lt.24848, 2-s2.0-85032260171. 29072825

[7] Namba M. , Hiramatsu A. , Aikata H. , Kodama K. , Uchikawa S. , Ohya K. , Morio K. , Fujino H. , Nakahara T. , Murakami E. , Yamauchi M. , Kawaoka T. , Tsuge M. , Imamura M. , and Chayama K. , Management of Refractory Ascites Attenuates Muscle Mass Reduction and Improves Survival in Patients With Decompensated Cirrhosis, Journal of Gastroenterology. (2020) 55, no. 2, 217–226, 10.1007/s00535-019-01623-4, 2-s2.0-85072184971, 31485782. 31485782

[8] Di Cola S. , Nardelli S. , Ridola L. , Gioia S. , Riggio O. , and Merli M. , Ammonia and the Muscle: An Emerging Point of View on Hepatic Encephalopathy, Journal of Clinical Medicine. (2022) 11, no. 3, 10.3390/jcm11030611. PMC8836353 35160063

[9] Tateyama M. , Naoe H. , Tanaka M. , Tanaka K. , Narahara S. , Tokunaga T. , Kawasaki T. , Yoshimaru Y. , Nagaoka K. , Watanabe T. , Setoyama H. , Sasaki Y. , and Tanaka Y. , Loss of Skeletal Muscle Mass Affects the Incidence of Minimal Hepatic Encephalopathy: A Case Control Study, BMC Gastroenterology. (2020) 20, no. 1, 10.1186/s12876-020-01501-x, 33167879. PMC7654593 33167879

[10] Nardelli S. , Lattanzi B. , Merli M. , Farcomeni A. , Gioia S. , Ridola L. , and Riggio O. , Muscle Alterations Are Associated With Minimal and Overt Hepatic Encephalopathy in Patients With Liver Cirrhosis, Hepatology. (2019) 70, no. 5, 1704–1713, 10.1002/hep.30692, 2-s2.0-85065841803, 31038758. 31038758

[11] Kim H. Y. and Jang J. W. , Sarcopenia in the Prognosis of Cirrhosis: Going Beyond the MELD Score, World Journal of Gastroenterology. (2015) 21, no. 25, 7637–7647, 10.3748/wjg.v21.i25.7637, 2-s2.0-84936792379. 26167066 PMC4491953

[12] Tantai X. , Liu Y. , Yeo Y. H. , Praktiknjo M. , Mauro E. , Hamaguchi Y. , Engelmann C. , Zhang P. , Jeong J. Y. , van Vugt J. L. A. , Xiao H. , Deng H. , Gao X. , Ye Q. , Zhang J. , Yang L. , Cai Y. , Liu Y. , Liu N. , Li Z. , Han T. , Kaido T. , Sohn J. H. , Strassburg C. , Berg T. , Trebicka J. , Hsu Y. C. , IJzermans J. N. M. , Wang J. , Su G. L. , Ji F. , and Nguyen M. H. , Effect of Sarcopenia on Survival in Patients With Cirrhosis: A Meta-Analysis, Journal of Hepatology. (2022) 76, no. 3, 588–599, 10.1016/j.jhep.2021.11.006, 34785325. 34785325

[13] Zeng X. , Shi Z. W. , Yu J. J. , Wang L. F. , Luo Y. Y. , Jin S. M. , Zhang L. Y. , Tan W. , Shi P. M. , Yu H. , Zhang C. Q. , and Xie W. F. , Sarcopenia as a Prognostic Predictor of Liver Cirrhosis: A Multicentre Study in China, Journal of Cachexia, Sarcopenia and Muscle. (2021) 12, no. 6, 1948–1958, 10.1002/jcsm.12797, 34520115. 34520115 PMC8718091

[14] Hirschfield G. M. and Gershwin M. E. , The Immunobiology and Pathophysiology of Primary Biliary Cirrhosis, Annual Review of Pathology: Mechanisms of Disease. (2013) 8, 303–330, 10.1146/annurev-pathol-020712-164014, 2-s2.0-84875890982. 23347352

[15] Selmi C. , Bowlus C. L. , Gershwin M. E. , and Coppel R. L. , Primary Biliary Cirrhosis, Lancet. (2011) 377, no. 9777, 1600–1609, 10.1016/S0140-6736(10)61965-4, 2-s2.0-79955689227. 21529926

[16] Floreani A. , Franceschet I. , Cazzagon N. , Spinazzè A. , Buja A. , Furlan P. , Baldo V. , and Gershwin M. E. , Extrahepatic Autoimmune Conditions Associated With Primary Biliary Cirrhosis, Clinical Reviews in Allergy and Immunology. (2015) 48, no. 2-3, 192–197, 10.1007/s12016-014-8427-x, 2-s2.0-84928995036. 24809534

[17] Trivedi H. D. , Danford C. J. , Goyes D. , and Bonder A. , Osteoporosis in Primary Biliary Cholangitis: Prevalence, Impact and Management Challenges, Clinical and Experimental Gastroenterology. (2020) 13, 17–24, 10.2147/CEG.S204638. 32021374 PMC6970242

[18] Lee C. M. , Kang B. K. , and Kim M. , Radiologic Definition of Sarcopenia in Chronic Liver Disease, Life. (2021) 11, no. 2, 10.3390/life11020086. PMC7910987 33504046

[19] Saeki C. , Saito M. , and Tsubota A. , Association of Chronic Liver Disease With Bone Diseases and Muscle Weakness, Journal of Bone And Mineral Metabolism. (2024) 42, no. 4, 399–412, 10.1007/s00774-023-01488-x. 38302761

[20] European Association for the Study of the Liver , EASL Clinical Practice Guidelines: The Diagnosis and Management of Patients With Primary Biliary Cholangitis, Journal of Hepatology. (2017) 67, no. 1, 145–172, 10.1016/j.jhep.2017.03.022, 2-s2.0-85017552637. 28427765

[21] European Association for the Study of the Liver , EASL 2017 Clinical Practice Guidelines on the Management of Hepatitis B Virus Infection, Journal of Hepatology. (2017) 67, no. 2, 370–398, 10.1016/j.jhep.2017.03.021, 2-s2.0-85017533115. 28427875

[22] Nishikawa H. , Shiraki M. , Hiramatsu A. , Moriya K. , Hino K. , and Nishiguchi S. , Japan Society of Hepatology Guidelines for Sarcopenia in Liver Disease (1st Edition): Recommendation From the Working Group for Creation of Sarcopenia Assessment Criteria, Hepatology Research. (2016) 46, no. 10, 951–963, 10.1111/hepr.12774, 2-s2.0-84985994790. 27481650

[23] Qiu M. L. , Xie Y. , Wang X. H. , Wang X. Q. , Zhao D. B. , Zhou H. Q. , Yan L. , Liang B. L. , Shen H. L. , and Cao S. Y. , Practice Guideline for Patients With Osteoporosis, Zhonghua Nei Ke Za Zhi. (2020) 59, no. 12, 953–959, 10.3760/cma.j.cn112138-20200904-00792. 33256336

[24] Wu M. C. and Meng Q. H. , Nutritional Assessment and Clinical Management of Patients With Acute-on-Chronic Liver Failure, Journal of Clinical Hepatology. (2021) 37, no. 4, 770–774.

[25] Hao R. R. , Wang H. , Wang H. Y. , Tang W. , and Lu Y. , Analysis of Risk Factors for Senile Sarcopenia and Its Relationship With NAFLD, Clinical and Experimental Medicine. (2020) 319, no. 15, 1588–1591.

[26] Meyer F. , Bannert K. , Wiese M. , Esau S. , Sautter L. F. , Ehlers L. , Aghdassi A. A. , Metges C. C. , Garbe L. A. , Jaster R. , Lerch M. M. , Lamprecht G. , and Valentini L. , Molecular Mechanism Contributing to Malnutrition and Sarcopenia in Patients With Liver Cirrhosis, International Journal of Molecular Sciences. (2020) 21, no. 15, 10.3390/ijms21155357, 32731496. PMC7432938 32731496

[27] Sinclair M. , Grossmann M. , Hoermann R. , Angus P. W. , and Gow P. J. , Testosterone Therapy Increases Muscle Mass in Men With Cirrhosis and Low Testosterone: A Randomised Controlled Trial, Journal of Hepatology. (2016) 65, no. 5, 906–913, 10.1016/j.jhep.2016.06.007, 2-s2.0-84995468329. 27312945

[28] Pan L. , Xie W. , Fu X. , Lu W. , Jin H. , Lai J. , Zhang A. , Yu Y. , Li Y. , and Xiao W. , Inflammation and Sarcopenia: A Focus on Circulating Inflammatory Cytokines, Experimental Gerontology. (2021) 154, 111544, 10.1016/j.exger.2021.111544. 34478826

[29] Ábrigo J. , Elorza A. A. , Riedel C. A. , Vilos C. , Simon F. , Cabrera D. , Estrada L. , and Cabello-Verrugio C. , Role of Oxidative Stress as Key Regulator of Muscle Wasting During Cachexia, Oxidative medicine and cellular longevity. (2018) 2018, no. 1, 2063179, 10.1155/2018/2063179, 2-s2.0-85052577509, 29785242. 29785242 PMC5896211

[30] Lai J. C. , Tandon P. , Bernal W. , Tapper E. B. , Ekong U. , Dasarathy S. , and Carey E. J. , Malnutrition, Frailty, and Sarcopenia in Patients With Cirrhosis: 2021 Practice Guidance by the American Association for the Study of Liver Diseases, Hepatology. (2021) 74, no. 3, 1611–1644, 10.1002/hep.32049, 34233031. 34233031 PMC9134787

[31] Dasarathy S. and Hatzoglou M. , Hyperammonemia and Proteostasis in Cirrhosis, Current Opinion in Clinical Nutrition and Metabolic Care. (2018) 21, no. 1, 30–36, 10.1097/MCO.0000000000000426, 2-s2.0-85036642310. 29035972 PMC5806198

[32] Dam G. , Ott P. , Aagaard N. K. , and Vilstrup H. , Branched-Chain Amino Acids and Muscle Ammonia Detoxification in Cirrhosis, Metabolic Brain Disease. (2013) 28, no. 2, 217–220, 10.1007/s11011-013-9377-3, 2-s2.0-84878016256. 23315357

[33] Davuluri G. , Allawy A. , Thapaliya S. , Rennison J. H. , Singh D. , Kumar A. , Sandlers Y. , van Wagoner D. R. , Flask C. A. , Hoppel C. , Kasumov T. , and Dasarathy S. , Hyperammonaemia-Induced Skeletal Muscle Mitochondrial Dysfunction Results in Cataplerosis and Oxidative Stress, Journal of Physiology. (2016) 594, no. 24, 7341–7360, 10.1113/JP272796, 2-s2.0-85003845721, 27558544. 27558544 PMC5157075

[34] Geraci A. , Calvani R. , Ferri E. , Marzetti E. , Arosio B. , and Cesari M. , Sarcopenia and Menopause: The Role of Estradiol, Frontiers In Endocrinology. (2021) 12, 682012, 10.3389/fendo.2021.682012. 34093446 PMC8170301

[35] Glass L. M. and Su G. L. , Metabolic Bone Disease in Primary Biliary Cirrhosis, Gastroenterology Clinics. (2016) 45, no. 2, 333–343, 10.1016/j.gtc.2016.02.009, 2-s2.0-84977083551. 27261902

[36] Liao C. Y. , Chung C. H. , Chu P. , Wei K. Y. , Feng T. M. , Lin F. H. , Tsao C. H. , Wu C. C. , and Chien W. C. , Increased Risk of Osteoporosis in Patients With Primary Biliary Cirrhosis, PLoS One. (2018) 13, no. 3, e0194418, 10.1371/journal.pone.0194418, 2-s2.0-85044027608, 29543880. 29543880 PMC5854410

[37] The Chinese Medical Association of Osteoporosis and Bone Mineral Disease, Zhang ZL. Guidelines for the Diagnosis and Treatment of Primary Osteoporosis (2022). Chinese, General Practice. (2023) 26, no. 14, 1671–1691.

[38] Li L. and Wang Z. , Ovarian Aging and Osteoporosis, Aging And Aging-Related Diseases: Mechanisms And Interventions. (2018) 1086, 199–215, 10.1007/978-981-13-1117-8_13, 2-s2.0-85053762156. 30232761

[39] Gu X. D. and Zhao D. B. , Research Progress of Osteoporosis Secondary to Primary Biliary Cholangitis, Journal of Osteoporosis. (2018) 24, no. 2, 276–280, 10.3748/wjg.v24.i31.3513, 2-s2.0-85052071679.

[40] Ruiz-Gaspà S. , Guañabens N. , Jurado S. , Dubreuil M. , Combalia A. , Peris P. , Monegal A. , and Parés A. , Bile Acids and Bilirubin Effects on Osteoblastic Gene Profile. Implications in the Pathogenesis of Osteoporosis in Liver Diseases, Gene. (2020) 725, 144167, 10.1016/j.gene.2019.144167, 31639434. 31639434

[41] Kirk B. , Al Saedi A. , and Duque G. , Osteosarcopenia: A Case of Geroscience, Aging Medicine. (2019) 2, no. 3, 147–156, 10.1002/agm2.12080. 31942528 PMC6880711

[42] Di Monaco M. , Castiglioni C. , Bardesono F. , Milano E. , and Massazza G. , Sarcopenia, Osteoporosis and the Burden of Prevalent Vertebral Fractures: A Cross-Sectional Study of 350 Women With Hip Fracture, European Journal of Physical And Rehabilitation Medicine. (2020) 56, no. 2, 184–190, 10.23736/S1973-9087.20.05991-2. 32052946

[43] Saeki C. , Oikawa T. , Kanai T. , Nakano M. , Torisu Y. , Sasaki N. , Abo M. , Saruta M. , and Tsubota A. , Relationship Between Osteoporosis, Sarcopenia, Vertebral Fracture, and Osteosarcopenia in Patients With Primary Biliary Cholangitis, European Journal of Gastroenterology And Hepatology. (2021) 33, no. 5, 731–737, 10.1097/MEG.0000000000001791, 32558699. 32558699 PMC8016510

[44] Chen L. K. , Woo J. , Assantachai P. , Auyeung T. W. , Chou M. Y. , Iijima K. , Jang H. C. , Kang L. , Kim M. , Kim S. , Kojima T. , Kuzuya M. , Lee J. S. W. , Lee S. Y. , Lee W. J. , Lee Y. , Liang C. K. , Lim J. Y. , Lim W. S. , Peng L. N. , Sugimoto K. , Tanaka T. , Won C. W. , Yamada M. , Zhang T. , Akishita M. , and Arai H. , Asian Working Group for Sarcopenia: 2019 Consensus Update on Sarcopenia Diagnosis and Treatment, Journal of the American Medical Directors Association. (2020) 21, no. 3, 300–307.e2, 10.1016/j.jamda.2019.12.012, 32033882. 32033882

[45] Campillo B. , Richardet J. P. , Scherman E. , and Bories P. N. , Evaluation of Nutritional Practice in Hospitalized Cirrhotic Patients: Results of a Prospective Study, Nutrition. (2003) 19, no. 6, 515–521, 10.1016/s0899-9007(02)01071-7, 2-s2.0-0038100248. 12781851

[46] European Association for the Study of the Liver , EASL Clinical Practice Guidelines on Nutrition in Chronic Liver Disease, Journal of Hepatology. (2019) 70, no. 1, 172–193, 10.1016/j.jhep.2018.06.024, 2-s2.0-85052084279. 30144956 PMC6657019

[47] Cheung K. , Lee S. S. , and Raman M. , Prevalence and Mechanisms of Malnutrition in Patients With Advanced Liver Disease, and Nutrition Management Strategies, Clinical Gastroenterology And Hepatology. (2012) 10, no. 2, 117–125, 10.1016/j.cgh.2011.08.016, 2-s2.0-84856054318. 21893127

[48] Cimsit C. , Kursun M. , Demircioglu O. , Dilber F. , Demirtas C. O. , and Ergenc I. , Radiological Quantification of Sarcopenic Obesity and Its Role in Chronic Liver Disease Severity, Academic Radiology. (2023) 30, no. supplement 1, S124–S131, 10.1016/j.acra.2023.03.001. 37012127

[49] Janmohamed A. and Trivedi P. J. , Patterns of Disease Progression and Incidence of Complications in Primary Biliary Cholangitis (PBC), Best Practice and Research Clinical Gastroenterology. (2018) 34, 71–83, 10.1016/j.bpg.2018.06.002, 2-s2.0-85049355443. 30343713

[50] Carrion A. F. , Lindor K. D. , and Levy C. , Safety of Fibrates in Cholestatic Liver Diseases, Liver International. (2021) 41, no. 6, 1335–1343, 10.1111/liv.14871. 33751787

[51] Corpechot C. , Chazouillères O. , Rousseau A. , Le Gruyer A. , Habersetzer F. , Mathurin P. , Goria O. , Potier P. , Minello A. , Silvain C. , and Abergel A. , A Placebo-Controlled Trial of Bezafibrate in Primary Biliary Cholangitis, New England Journal of Medicine. (2018) 378, no. 23, 2171–2181, 10.1056/NEJMoa1714519, 2-s2.0-85048328032, 29874528. 29874528

[52] Langford N. J. and Kendall M. J. , Rhabdomyolysis With HMG CoA Reductase Inhibitors: A Class Effect?, Journal of Clinical Pharmacy and Therapeutics. (2001) 26, no. 6, 391–395, 10.1046/j.1365-2710.2001.00369.x, 2-s2.0-0035725924. 11722675

[53] Kowdley K. V. , Bowlus C. L. , Levy C. , Akarca U. S. , Alvares-da-Silva M. R. , Andreone P. , Arrese M. , Corpechot C. , Francque S. M. , Heneghan M. A. , Invernizzi P. , Jones D. , Kruger F. C. , Lawitz E. , Mayo M. J. , Shiffman M. L. , Swain M. G. , Valera J. M. , Vargas V. , Vierling J. M. , Villamil A. , Addy C. , Dietrich J. , Germain J. M. , Mazain S. , Rafailovic D. , Taddé B. , Miller B. , Shu J. , Zein C. O. , and Schattenberg J. M. , Efficacy and Safety of Elafibranor in Primary Biliary Cholangitis, New England Journal of Medicine. (2024) 390, no. 9, 795–805, 10.1056/NEJMoa2306185. 37962077

